# Identifying pleiotropic genes in genome-wide association studies from related subjects using the linear mixed model and Fisher combination function

**DOI:** 10.1186/s12859-017-1791-9

**Published:** 2017-08-24

**Authors:** James J. Yang, L Keoki Williams, Anne Buu

**Affiliations:** 1 School of Nursing, University of Michigan, Ann Arbor, 48104 Michigan USA; 2 Department of Internal Medicine, Henry Ford Health System, Detroit, 48202 Michigan USA; 3 The Center for Health Policy and Health Services Research, Henry Ford Health System, Detroit, 48202 Michigan USA; 4 Department of Health Behavior and Biological Sciences, University of Michigan, Ann Arbor, 48104 Michigan USA

**Keywords:** Genome-wide association study, Fisher combination function, Pleiotropy, Alcohol dependence, Substance abuse

## Abstract

**Background:**

A multivariate genome-wide association test is proposed for analyzing data on multivariate quantitative phenotypes collected from related subjects. The proposed method is a two-step approach. The first step models the association between the genotype and marginal phenotype using a linear mixed model. The second step uses the correlation between residuals of the linear mixed model to estimate the null distribution of the Fisher combination test statistic.

**Results:**

The simulation results show that the proposed method controls the type I error rate and is more powerful than the marginal tests across different population structures (admixed or non-admixed) and relatedness (related or independent). The statistical analysis on the database of the Study of Addiction: Genetics and Environment (SAGE) demonstrates that applying the multivariate association test may facilitate identification of the pleiotropic genes contributing to the risk for alcohol dependence commonly expressed by four correlated phenotypes.

**Conclusions:**

This study proposes a multivariate method for identifying pleiotropic genes while adjusting for cryptic relatedness and population structure between subjects. The two-step approach is not only powerful but also computationally efficient even when the number of subjects and the number of phenotypes are both very large.

## Background

After the completion of the Human Genome Project [1] and a successful case-control experiment in identifying age-related markers using single-nucleotide polymorphism (SNP) [2], the number of genome-wide association study (GWAS) has been rising exponentially [3]. GWAS provides an efficient way to scan the whole genome to locate SNPs associated with the trait of interest which may potentially lead to identification of the susceptibility gene through linkage disequilibrium. Unlike linkage analysis that requires data collection from genetically related subjects, GWAS is applicable to a more general setting involving independent subjects. This makes GWAS highly desirable because for many diseases, it may not be feasible to recruit enough related subjects for linkage analysis. For example, the parents of human subjects with late onset diseases are usually not available. Furthermore, many statistical programs such as PLINK [4] have been developed to manage and analyze high dimensional GWAS data from *independent subjects*.

Due to reduced costs for SNP arrays, in recent years, many family studies have collected GWAS data [5–7]. If existing methods designed for independent subjects are adopted to analyze these data, the power of association tests will be greatly reduced because only a subset of data can be used. On the other hand, employing all the data in the analysis (i.e. ignoring the correlation between genetically related subjects) may result in false positive findings [8]. Yu et al. (2006) [9] proposed a compromise between these two approaches that used all related subjects while adjusting for the relatedness by random effects in a linear mixed model. This approach has been widely studied and the original algorithm has been improved to be applicable to larger scale studies [10]. However, this approach can only handle a *univariate phenotype* such as a positive or negative diagnosis.

Many complex diseases such as mental health disorders have multiple phenotypic traits that are correlated [11]. These multivariate phenotypes may point to a shared genetic pathway and underscore the relevance of pleiotropy (i.e., a gene or genetic variant that affects more than one phenotypic trait, Solovieff et al. (2013) [12]). Furthermore, a statistical model searching for loci that are simultaneously associated with multiple phenotypes has higher power than a model that only considers each phenotype individually [13]. Our research team recently developed a multivariate association test based on the Fisher combination function that can be applied to analyze GWAS data with multivariate phenotypes [14]. This method, however, can only handle *independent subjects*. Taken together, advanced methods that can handle multivariate phenotypes and related subjects *simultaneously* are highly desirable.

The crucial problem in GWAS is to deal with confounders such as population structure, family structure, and cryptic relatedness. Astle and Balding [15] reviewed approaches to correcting association analysis for confounding factors. When family-based samples are collected, analysis based on the transmission disequilibrium test is robust to population structure. Several methods have been developed for multivariate phenotype data collected from family-based samples [16–20]. However, the major challenge of this type of studies is to recruit enough families in order to conduct the analysis with sufficient power. This type of studies also have limited applications to late-onset diseases. Recently, Zhou and Stephens [21] proposed a multivariate linear mixed model (mvLMMs) for identifying pleiotropic genes. This approach can handle a mixture of unrelated and related individuals and thus has broader applications. However, it was recommended for a modest number of phenotypes (less than 10) due to computational and statistical barriers of the EM algorithm [21].

For related subjects with multivariate phenotypes, there are two sources of correlations between multivariate phenotypes: one is the correlation arising from genetically related subjects whose phenotypes are more highly correlated because of shared genotypes; and the other is the correlation between multiple phenotypes which exists even when independent subjects are employed. This study proposes a new statistical method that can model both sources of correlations. We also compare the performance of the proposed method with that of the mvLMMs method. The rest of this paper is organized as follows. In the “Methods” section, we review our previous work on multivariate phenotypes in independent subjects and also extend the method to handle related subjects. The “Results” section summaries the results of simulation studies and statistical analysis on the Study of Addiction: Genetics and Environment (SAGE) data. Future directions and major findings are presented in “Discussion” and “Conclusions” sections, respectively.

## Methods

Suppose that for each subject, we measure *R* different phenotypes and run an assay with *S* SNPs. The resulting measurements can be organized with two data matrices. The genotype data are stored in a *S*×*N* matrix where *N* is the total number of subjects and each element of the matrix is coded as 0, 1, or 2 copies of the reference allele. The phenotype data are stored in an *N*×*R* matrix where each row records the individual’s multivariate phenotypes. Studying the association between genotypes and phenotypes, thus, involves measuring and testing the association between each row of the genotype matrix and the entire phenotype matrix. Since one SNP is consider at a time, the association test is repeated *S* times for all SNPs. Specifically, Let *β*_1_,…,*β*_*R*_ be the effect sizes of a candidate SNP on *R* different phenotypes. The null hypothesis of testing the pleiotropic gene is

$$H_{0}: \beta_{1}= \ldots = \beta_{R} = 0. $$

If this *H*_0_ is rejected, we claim that the corresponding SNP is associated with the pre-determined multivariate phenotypes.

When the phenotype is univariate, the association test for GWAS data can be carried out using commonly adopted software such as R [22] or PLINK [4]. For multivariate phenotypes in independent subjects, Yang et al. (2016) [14] has conducted a comprehensive review of various multivariate methods and proposed a method using the Fisher combination function. They further showed that their proposed method is better than other existing methods. The following sections briefly review their method and extend it to handle related subjects by employing a linear mixed model to adjust for relatedness.

### Review of previous work on independent subjects with multivariate phenotypes

To illustrate the method proposed by Yang et al. (2016) [14], we define the notations for genotypes and phenotypes. Let *i*(=1,…,*N*) be the index of individuals. Define $y^{r}_{i}$ as the *r*th phenotype of individual *i* (*r*=1,…,*R*) and $g^{s}_{i}$ as the *s*th genotype of individual *i* (*s*=1,…,*S*). Therefore, the vector $\boldsymbol {y}^{r} = (y^{r}_{1},\ldots,y^{r}_{N})$ represents the *r*th marginal phenotype collected from *N* individuals and the vector $\boldsymbol {g}^{s} = (g^{s}_{1},\ldots,g^{s}_{N})$ represents the genotypes of the *s*th SNP from *N* individuals.

When *R*=1 (i.e., the phenotype is univariate), a regression model is commonly adopted to model ***y*** ^*r*^ as a function of ***g*** ^*s*^ with covariates in the model to adjust for confounding factors or to increase the precision of estimates. When *R*>1 (i.e., multivariate phenotypes), Yang et al. (2016) [14] proposed a two-step approach. In the first step, for each phenotype *r*, a marginal *p*-value, *p*_*rs*_, is derived from a likelihood ratio test under a linear regression of ***y*** ^*r*^ on ***g*** ^*s*^. The next step is to test association between *R* multivariate phenotypes and the *s*th SNP based on these marginal *p*-values of *p*_1*s*_,…,*p*_*Rs*_. The Fisher combination function is used to combine them and the test statistic is defined as

1 $$ \xi_{s} = \sum_{r=1}^{R} -2\log (p_{rs}).  $$

The SNP *s* is claimed to be associated with the *R* multivariate phenotypes if *ξ*_*s*_ is statistically significant. Although −2 log(*p*_*rs*_) follows a chi-square distribution with 2 degrees of freedom, *ξ*_*s*_, which is a sum of *dependent* chi-square random variables, does not follow a chi-square distribution with 2*R* degrees of freedom. The permutation method may be adopted to calculate the *p*-value of *ξ*_*s*_ but it is computationally too expensive in the context of GWAS (see Yang et al. (2016) [14] for details).

Under the the null hypothesis, the statistic *ξ*_*s*_ is the sum of dependent chi-square statistics. Thus, the null distribution of *ξ*_*s*_ follows a gamma distribution [23, 24] with the mean and variance being functions of the shape parameter *k* and the scale parameter *θ*:

$$\begin{array}{*{20}l} E[\xi_{s}] &= k\theta,\\ Var[\xi_{s}] &= k \theta^{2}. \end{array} $$

Applying the method of moments, we can derive the following equations based on the first two sample moments:

2 $$\begin{array}{*{20}l} k\theta &= 2R, \end{array} $$

3 $$\begin{array}{*{20}l} k \theta^{2} &= 4R + \sum_{r\neq r'} cov(-2\log (p_{rs}), -2\log (p_{r's})). \end{array} $$

Yang et al. (2016) [14] showed that the pairwise sample correlation $\rho _{rr'}=\text {cor}(\boldsymbol {y}^{r},\boldsymbol {y}^{r'})\phantom {\dot {i}\!}$ can be used to accurately estimate $cov(-2\log (p_{rs}), -2\log (p_{r's}))\phantom {\dot {i}\!}$ as follows:

4 $$ {}cov\left(-2\log (p_{rs}), -2\log (p_{r's})\right) \approx \sum_{l=1}^{5} c_{l} \rho_{rr'}^{2l} - \frac{c_{1}}{N} \left(1-\rho_{rr'}^{2}\right)^{2}\!,  $$

where *c*_1_=3.9081, *c*_2_=0.0313, *c*_3_=0.1022, *c*_4_=−0.1378 and *c*_5_=0.0941. This approximation is very accurate as the maximum difference is less than 0.0001. Thus, we can efficiently estimate *k* and *θ* using Eqs. (2) and (3) with the *cov*(·) in Eq. (3) substituted by the right-hand side of Eq. (4).

### The proposed method for related subjects with multivariate phenotypes

The multivariate method described in the previous section only applies to independent subjects. When multivariate phenotypes data are collected from genetically related subjects, there are two types of correlations: 1) the correlation between multivariate phenotypes (even when the subjects are independent); and 2) the correlation due to genetically related subjects (even when the phenotype is univariate). The approach described in the previous section only addresses the first type of correlation. To address both correlations in the regression model, the marginal regression model in the first step needs to be modified to account for genetically related subjects. Recall that the original regression model has the form of

$$ \boldsymbol{y}^{r} = \boldsymbol{\alpha}^{r} + \boldsymbol{g}^{s} \boldsymbol{\beta}^{r} + \boldsymbol{\epsilon}^{r}, $$

where ***α*** ^*r*^ is the intercept term, ***β*** ^*r*^ is the genetic effect and ***ε*** ^*r*^∼*N*(0,*σ* ^2^ ***I***) is a vector of error terms. When the subjects are genetically related, we modify the model to be a linear mixed model:

5 $$ \boldsymbol{y}^{r} = \boldsymbol{\alpha}^{r} + \boldsymbol{g}^{s} \boldsymbol{\beta}^{r} + \boldsymbol{z}^{r}+\boldsymbol{\epsilon}^{r},   $$

where the added term ***z*** ^*r*^ is a random effect and it follows $N(0,\sigma _{g}^{2} \boldsymbol {K})$ where the matrix ***K*** is called the genetic relationship matrix (GRM) [25].

Direct calculation of the best linear unbiased estimates of the fixed effects and the best linear unbiased predictors of the random effects for a large sample size is extremely slow and may be beyond the memory capacity of most computers. Many flexible and efficient methods have been developed to carry out GWAS using linear mixed models. For example, the efficient algorithm implemented in GCTA [25] uses the restricted maximum likelihood (REML) method to estimate $\sigma _{g}^{2}$ and *σ* ^2^ under the null model while the GRM ***K*** was estimated from all the SNPs. To test *H*_0_:***β*** ^*r*^=0, the estimates of the random effects ($\sigma _{g}^{2}$, *σ* ^2^, and ***K***) under the null model were plugged in for the estimation of the variance of the $\boldsymbol {\hat {\beta }}^{r}$. In this way, the Wald test statistic can be constructed. Under *H*_0_, this statistic follows an asymptotical chi-squared distribution with 1 degree of freedom; and the corresponding marginal *p*-value indicates the strength of association between the SNP and a marginal phenotype.

The resulting marginal *p*-values, *p*_1*s*_,…,*p*_*Rs*_, can then be combined together using the Fisher combination function in Eq. (1) to form the test statistic *ξ*_*s*_ for the association between the *s*th SNP and *R* multivariate phenotypes. Based on the linear mixed model in Eq. (5), it can be shown that for different traits ***y*** ^*r*^ and $\phantom {\dot {i}\!}\boldsymbol {y}^{r'}$, the covariance between them is

$$\text{cov}\left(\boldsymbol{y}^{r}, \boldsymbol{y}^{r'}\right) =\nu\sigma_{g}^{2} \boldsymbol{K} + \rho_{rr'} \sigma^{2} I, $$

where *ν* is the genetic correlation due to related subjects and $\phantom {\dot {i}\!}\rho _{rr'}$ is the correlation between phenotypes (even when only independent subjects are involved). Because the test statistic *ξ*_*s*_ is a function of *p*_1*s*_,…,*p*_*Rs*_ which are derived with the relatedness between subjects being adjusted by the random effect ***z*** ^*r*^ in Model (5), we can use the pairwise correlation between residuals, $\phantom {\dot {i}\!}\text {cor}(\boldsymbol {\epsilon }^{r}, \boldsymbol {\epsilon }^{r'})$, from this model to estimate $\phantom {\dot {i}\!}\rho _{rr'}$ and plug this estimate into Eq. (4). In this way, the null distribution of *ξ*_*s*_ can be approximated.

Although the GRM associated with the random effect ***z*** ^*r*^, in principle, contains information about the population structure resulting from systematic differences in ancestry, the random effect is not likely to be estimated perfectly in practice. For this reason, we proposed to extend the linear mixed model in Eq. (5) by adding principal components [26] estimated from genotype data as covariates for the purpose of improving the precision of the estimates for marginal *p*-values. This was based on the results of Astle el al. (2009) [15] showing that combining GRM with principal components could account for the population structure and relatedness better. Because contemporary American genomes resulted from a sequence of admixture process involving individuals descended from multiple ancestral population groups [27], this additional adjustment may potentially be a crucial step and its effectiveness was evaluated through simulation studies described in the next section.

## Results

### Simulation studies

#### Generating the genotype data

We simulated genotype data based on two different population structures (parents from the same population or parents from two different populations) and two different relatedness structures (independent subjects or related subjects) so there were four different types of data sets reflecting all possible combinations. We generated a set of allele frequencies (corresponding to a total of 10,000 SNPs) from uniform random numbers between 0.1 and 0.9 to represent Population I; and another set of allele frequencies to represent Population II. Given a set of population allele frequencies, we can generate the genotypes of parents from the particular population. Through random mapping, we can generate three types of parents (1/3 each): (1) both parents from Population I; (2) both parents from Population II; and (3) one parent from Population I and the other parent from Population II.

Once we had simulated parents’ genotypes, the genes were dropped down the pedigree according to Mendel’s law to simulate children’s genotypes. Our procedure ensured that children from different families represented independent subjects and children within a family represented strongly related subjects. To generate a sample of independent subjects, we simulated 1000 families with one child from each family. To generate a sample of related subjects, we simulated 250 families with four children in each family. Depending on whether parents’ genotypes were generated from one population (either Population I or Population II) or from two different populations, we had four scenarios of children genotype samples: 1) independent samples from non-admixed/isolated population (Non-admixed Independent); 2) related samples from non-admixed/isolated population (Non-admixed Related); 3) independent samples from admixed population (Admixed Independent); and 4) related samples from admixed population (Admixed Related).

#### Evaluating the phenotype correlation estimates

To evaluate the methods for estimating the correlation between phenotypes $\phantom {\dot {i}\!}\rho _{rr'}$, we simulated bivariate phenotypes using bivariate normal (BVN) random variables. An additive genetic effect was used to model the relationship between the genotype and bivariate phenotypes. Let *e* be the genetic effect size. The mean value of the marginal phenotype *μ*_*r*_(*r*=1,2) was −*e* if the genotype was *AA*; 0 if the genotype was *AB*; and *e* if the genotype was *BB*. The specific model to simulate phenotypes is:

6 $$ \left(\begin{array}{c} Y_{1}\\ Y_{2} \end{array}\right) \sim \text{BVN } \left(\left(\begin{array}{c} \mu_{1}\\ \mu_{2} \end{array}\right), \Sigma \right),   $$

where *Σ* is a 2×2 symmetric matrix with the diagonal elements being 1 and the off-diagonal element *ρ*. The value of *ρ* determines the correlation between the phenotypes. For each data set, the values of *ρ* ranged from 0 (independent) to 0.9 (highly dependent), and the values of *e* ranged from 0 (no effect) to 1 (large effect).

Each configuration was simulated 1000 times. In each simulated data set, we calculated the estimate for $\phantom {\dot {i}\!}\rho _{rr'}$ based on three methods: Method 1: the residuals from the linear mixed model; Method 2: the residuals from the linear mixed model with the first ten principal components as covariates; Method 3: the correlation between simulated phenotypes.

The third one was a näive method that did not adjust for the correlation due to related subjects and thus was expected to overestimate $\phantom {\dot {i}\!}\rho _{rr'}$. The program GCTA was used to fit linear mixed models and calculate corresponding residuals.

**Fig. 1:**
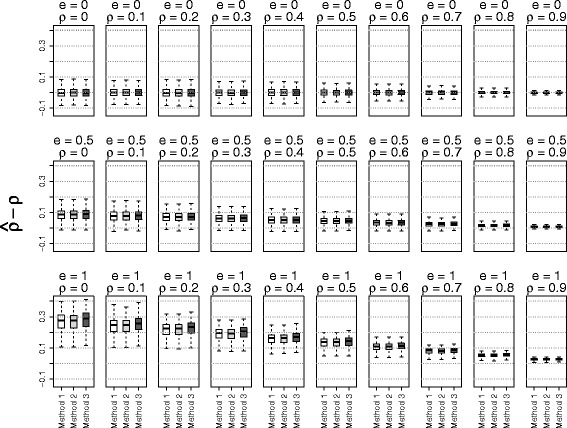
The accuracy of correlation estimations based on three methods for data from *non-admixed independent* subjects (Method 1: linear mixed model; Method 2: linear mixed model with principal components; Method 3: correlation without adjusting for relatedness)

**Fig. 2:**
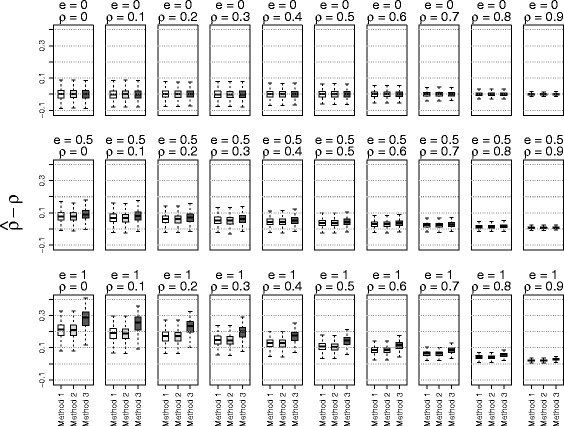
The accuracy of correlation estimations based on three methods for data from *non-admixed related* subjects (Method 1: linear mixed model; Method 2: linear mixed model with principal components; Method 3: correlation without adjusting for relatedness)

**Fig. 3:**
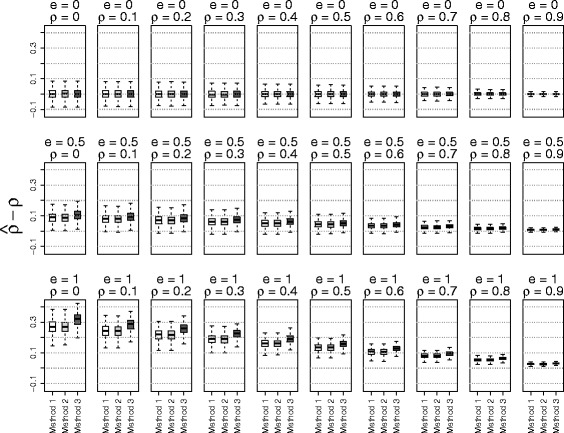
The accuracy of correlation estimations based on three methods for data from *admixed independent* subjects (Method 1: linear mixed model; Method 2: linear mixed model with principal components; Method 3: correlation without adjusting for relatedness)

**Fig. 4:**
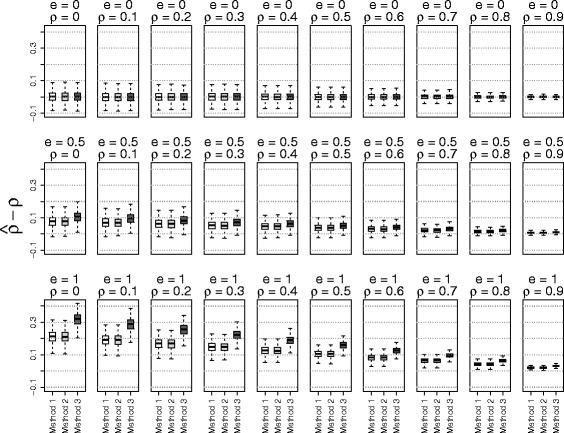
The accuracy of correlation estimations based on three methods for data from *admixed related* subjects (Method 1: linear mixed model; Method 2: linear mixed model with principal components; Method 3: correlation without adjusting for relatedness)

The simulation results based on the three methods of estimating $\phantom {\dot {i}\!}\rho _{rr'}$ were shown in Figs. 1, 2, 3 to 4 using boxplots to represent the distribution of $\hat {\rho } - \rho $. A good method was identified by choosing the one with the mean values of $\hat {\rho } - \rho $ close to zero. Comparing the accuracies of these three methods, it shows that the accuracy of estimation depended on the values of the true correlation and effect size. When the effect size *e* was 0 (no effect) or when the phenotype correlation was highly correlated (near 0.9), all three methods performed well. On the other hand, when the effect size was large (*e*=1) and the phenotype correlation was small (near 0), all three methods over estimated the true correlation. However, in this situation, the methods using residuals from linear mixed models performed better than the näive method. To our surprise, adding principal components in the linear mixed model did not substantially improve the accuracy of estimates. Because of the poor performance of the näive method, it was not used for the simulations evaluating the type I error rate and power.

#### Evaluating the type I error and power of the proposed method

Because the proposed method was designed to identify pleiotropic genes, evaluating the performance of the multivariate association test in terms of the type I error and power is essential. We simulated four correlated phenotypes using multivariate normal (MVN) random variables. The values of the genetic effect size, *e*, were 0 (no effect), 0.1 (medium effect), and 0.2 (large effect) and the values of the correlation *ρ* were 0 (independent), 0.4 (moderate correlated), and 0.8 (highly correlated). Each configuration was repeated 1000 times.

**Fig. 5:**
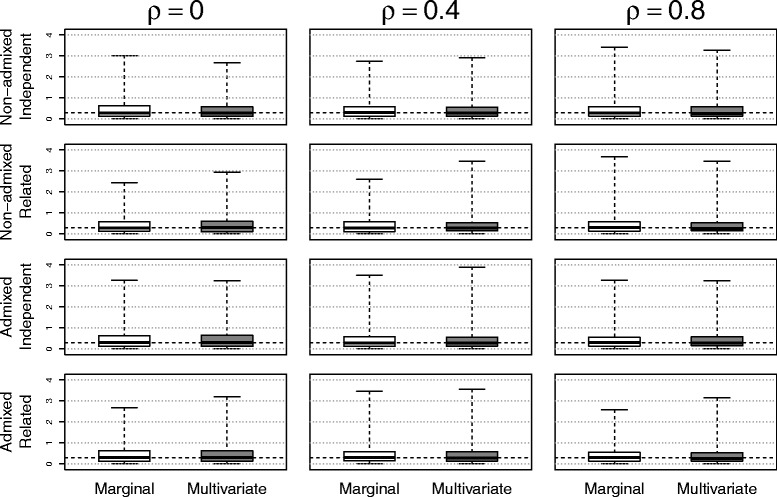
The distribution of values of − log10(*p*) using samples with different population structures and relatedness for various correlations *ρ* between phenotypes under the null hypothesis: the effect size *e*=0. The white boxes correspond to the marginal test; and the gray boxes correspond to the multivariate test

**Fig. 6:**
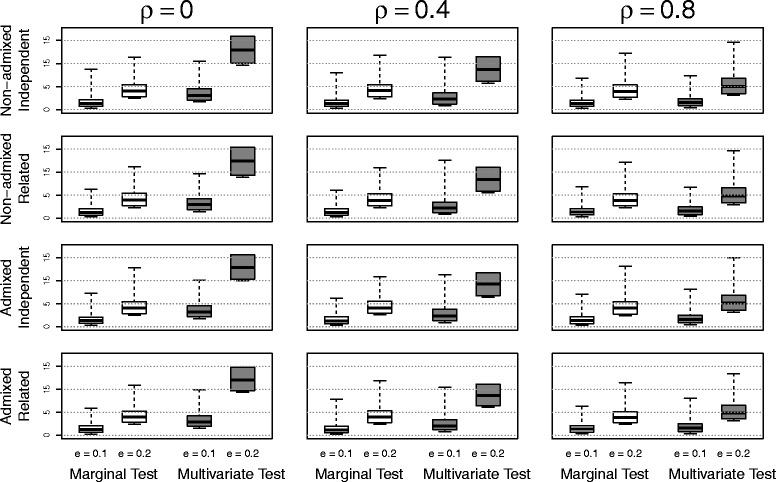
The distribution of values of − log10(*p*) using samples with different population structures and relatedness for various correlations *ρ* between phenotypes under the alternative hypotheses: the effect size *e*=0.1,0.2. The white boxes correspond to the marginal test; and the gray boxes correspond to the multivariate test

Figures 5 and 6 shows the distribution of − log10(*p*) for different values of correlation *ρ* and genetic effect size *e*. Large values of − log10(*p*)-value are equivalent to small *p*-values. Thus, when the effect size was large, we expected − log10(*p*) to be large. Based on our configuration to generate phenotypes, there was no difference between the four marginal *p*-values. Hence, we only presented the distribution of marginal *p*-values corresponding to the first marginal phenotype. The multivariate *p*-values were derived using the proposed method with the correlation estimated by the residuals from the linear mixed model with the first ten principal components as covariates. The findings from this simulation study were summarized as follows:

1. When there was no genetic effect (*e*=0), both the marginal and multivariate methods produced uniform *p*-values distributions which reflected the null distribution of *p*-values. When the genetic effect size increased, the value of − log10(*p*) increased. Therefore, the simulation showed that both marginal and multivariate tests were unbiased.
2. When the population structure and relatedness were fixed, increasing the correlation between phenotypes decreased the power of multivariate tests. The negative relationship between the correlation of multivariate phenotypes and power has also been observed in Yang et al. (2016) for various multivariate testing statistics [14].
3. When the genetic effect was not zero, the proposed multivariate method was more powerful than the marginal test in all situations. The advantages of using the multivariate method was most evident when the correlation between phenotypes was small to moderate. But even when the correlation between phenotypes was as large as 0.8, the multivariate method was still more powerful than the marginal tests. Therefore, combining multivariate phenotypes could increase the power of test.
4. When the sample size was held constant (recall that the sample size was the same across different population structure and relatedness in our simulation), the difference in power between admixed and non-admixed samples or between independent or related samples were very small.

#### Comparing the proposed method with the mvLMMs method

We further evaluated the performance of the proposed method in comparison to a competing method, the multivariate linear mixed model (mvLMMs) method, that has been implemented in the GEMMA [28] software. Here, we adopted the most complex situation from the previous simulation experiment in which genotypes were simulated based on related people from admixed populations (Admixed Related). Specifically, we simulated genotypes from 250 families each of which had four children and resulted in 1000 related individuals. Next, we simulated the following phenotypes from these genotypes by extending Model (6) to

$$\left(\begin{array}{c} Y_{1}\\ \vdots\\ Y_{4} \end{array}\right) \sim \text{BVN } \left(\left(\begin{array}{c} \mu_{1}\\ \vdots\\ \mu_{4} \end{array}\right), \Sigma \right), $$

where *Σ* was a 4×4 symmetric matrix with the diagonal elements being 1 and the off-diagonal element *ρ*. We manipulated the value of *ρ* to be 0.1 (weak correlated) or 0.5 (moderate correlated). Let ***e***=(*e*_1_,…,*e*_4_) be the genetic effect sizes corresponding to the phenotypes *Y*_1_,…,*Y*_4_. We considered the following combinations:

1. Small effect sizes: ***e***=(0.1,0.1,0.1,0.1);
2. Increasing effect sizes: ***e***=(0.05,0.1,0.15,0.2);
3. Medium effect sizes: ***e***=(0.15,0.15,0.15,0.15).

We did not consider the situation of no effect (i.e., ***e***=(0,0,0,0)) because both methods have been shown to control the type I error.

We simulated each configuration 1000 times. For our proposed method, we estimated pairwise correlation $\phantom {\dot {i}\!}\rho _{rr^{\prime }}$ based on **Method 1** described in the previous section for its good performance.

**Fig. 7:**
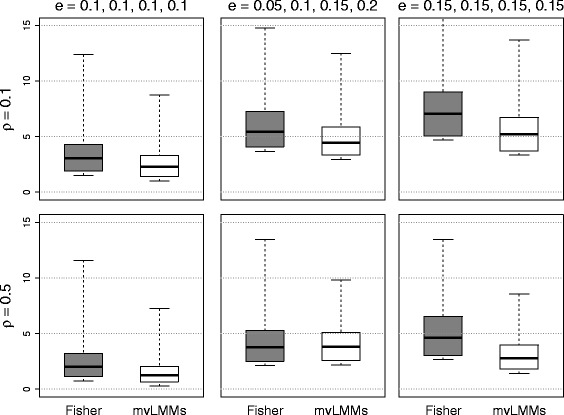
Comparing the power of two competing methods: the distribution of values of − log10(*p*) under different correlations *ρ* and effect sizes *e*. The gray boxes correspond to the proposed Fisher method; and the white boxes correspond to the mvLMMs method

Figure 7 shows the distribution of − log10(*p*) for different values of the correlation *ρ* and the genetic effect sizes ***e***. A powerful method should result in small *p*-values (or equivalently, large values of − log10(*p*)). The findings form this simulation study were summarized as follows:

1. The power of both methods depended on the effect sizes. When the effect sizes were increased from small to medium, the power of both methods increased. More importantly, the scale of such increase was larger for the proposed method.
2. When the effect sizes were fixed, both methods had higher power when the correlation between phenotypes was weak.
3. When the effect sizes were not equal among marginal phenotypes, the proposed method still maintained its high performance.
4. Overall, the proposed method was more powerful than the mvLMMs method. The proposed method had a larger median value of − log10(*p*) compared to the mvLMMs method in 5 our of the 6 configurations. The mvLMMs only achieved the same level of performance when the phenotypes had a medium correlation and the effect sizes were increasing.

In addition to high power, the proposed method has the advantage of being computationally efficient even when the number of phenotypes is large. The mvLMMs method, on the other hand, was recommended for a modest number of phenotypes (less than 10) due to computational and statistical barriers of the EM algorithm [21].

### Real data analysis

We demonstrate the application of the proposed method by conducting analysis on the data from the Study of Addiction: Genetics and Environment (SAGE).

The SAGE is a study that collected data from three large scale studies in the substance abuse field: the Collaborative Study on the Genetics of Alcoholism (COGA), the Family Study of Cocaine Dependence (FSCD), and the Collaborative Genetic Study of Nicotine Dependence (COGEND). The total number of subjects in all three studies was 4121. Each subject was genotyped using the Illumina Human 1M-Duo beadchip which contains over 1 million SNP markers. From the original 4121 individuals, some subjects were genotyped twice so we eliminated duplicate samples and the sample size was reduced to be 4112. Although dbGap provided a PED file to show pedigree and relationship among participants, we used the KING program [29] to verify their relationship. As a result, we confirmed and identified 3921 unrelated individuals and the remaining 191 were family members of these unrelated individuals. Using the chosen 4112 individuals, we restricted SNPs to 22 autosomes and conducted quality control of SNPs based on the minor allele frequency (>0.01), Hardy-weinberg equilibrium test (*p*-value >10^−5^), and frequency of missingness per SNP (<0.05) [30]. The final total number of SNPs chosen for analysis was 711,038.

Because our research aimed to identify the SNPs associated with the risk for alcohol dependence, four correlated phenotypes were used for the analysis:

1. age_first_drink:
  the age when the participant had a drink containing alcohol the first time.
2. ons_reg_drink: the onset age of regular drinking (drinking once a month for 6 months or more).
3. age_first_got_drunk:
  the age when the participant got drunk the first time.
4. alc_sx_tot: the number of alcohol dependence symptoms endorsed.

**Fig. 8:**
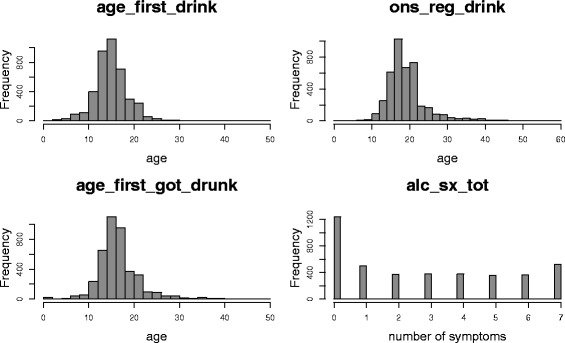
The distributions of four phenotypes indicating the risk for alcohol dependence using real data from 4121 participants

To deal with missing values in any of these four phenotypes, we imputed them using the mi package [31] from R software. The sample distributions of phenotypes and their pairwise correlations are shown in Fig. 8 and Table 1, respectively. The first three variables are the onset ages of important “milestone” events of alcoholism. Earlier onset ages are indicators for higher vulnerability and have been shown to predict later progression to alcohol dependence [32]. Thus, they were expected to be positively correlated with each other and negatively correlated with the number of alcohol dependence symptoms.

**Table 1:** The correlations among the 4 alcohol dependence phenotypes

Correlation	age_first_drink	ons_reg_drink	age_first_got_drunk	alc_sx_tot
age_first_drink	1.00	0.47	0.60	-0.47
ons_reg_drink	0.47	1.00	0.55	-0.26
age_first_got_drunk	0.60	0.55	1.00	-0.30
alc_sx_tot	-0.47	-0.26	-0.30	1.00

**Fig. 9:**
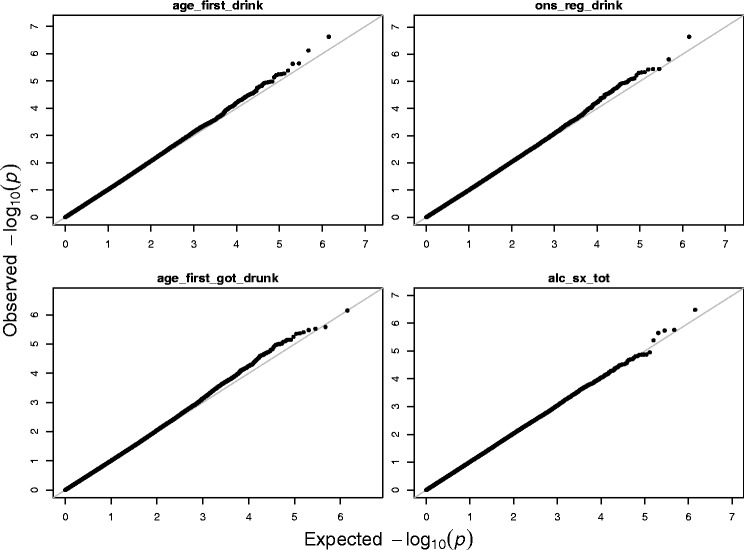
The QQ-plots of − log10(*p*)-values from marginal tests for four alcohol dependence phenotypes using real data from 4121 participants

**Fig. 10:**
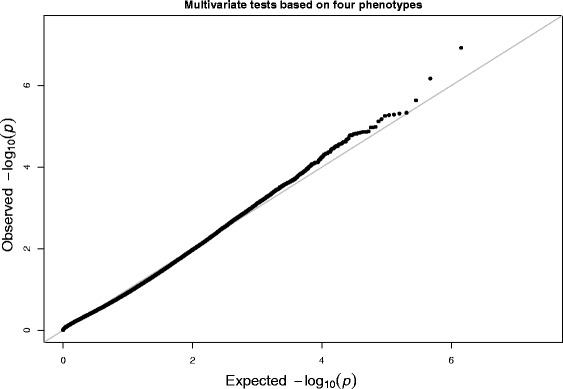
The QQ-plot of − log10(*p*)-values from the multivariate test for four alcohol dependence phenotypes using real data from 4121 individuals

We conducted marginal genome-wide association tests on each of these four phenotypes using the GCTA program to account for relatedness among subjects. We also added the first ten principal components to increase the precision of estimates. In addition to these principal components, the participant’s gender, age at interview, and self-identified race were included as covariates in the model. The regression model for the marginal phenotype is

$$ \boldsymbol{y}^{r} = \boldsymbol{\alpha}^{r} + \boldsymbol{x} \boldsymbol{\eta}^{r}+ \boldsymbol{g}^{s} \boldsymbol{\beta}^{r} + \boldsymbol{z}^{r}+\boldsymbol{\epsilon}^{r}, $$

where ***x*** contains the participant’s first ten principal components, gender, age at interview, and self-identified race, and the corresponding regression coefficients ***η*** ^*r*^ are treated as fixed effects. The QQ-plots of the *p*-values for the marginal association tests are shown in Fig. 9. The QQ-plot of the *p*-values for the proposed multivariate tests is displayed in Fig. 10. Since a primary assumption in GWAS is that most SNPs are not associated with the phenotype studied, most points in the QQ-plots should not deviate from the diagonal line. Deviations from the diagonal line may indicate that potential confounders such as the population structure or relatedness are not adjusted. Both of Figs. 9 and 10 indicate that potential confounders were well adjusted in our real data analysis.

To identify the SNPs associated with the risk for alcohol dependence, we declared significant SNPs if their *p*-values were less than the significance level of 10^−6^. Based on the marginal tests, two SNPs (*rs*9825428, *p*-value =2.3962×10^−7^; *rs*16822575, *p*-values =7.6411×10^−7^) were associated with age_first_drink; one SNP (*rs*11157640, *p*-value =2.2658×10^−7^) was associated with ons_reg_drink; one SNP (*rs*7700665, *p*-value =7.0618×10^−7^) was associated with age_first_got_drunk; and one SNP (*rs*10914375, *p*-value =3.2978×10^−7^) was associated with alc_sx_tot. On the other hand, using the proposed multivariate method, two SNPs (*rs*7523645, *p*-value =1.1872×10^−7^; and *rs*11157640, *p*-value =6.6655*e*×10^−7^) were significantly associated with these four correlated phenotypes for the risk of alcohol dependence.

Based on the findings in the marginal tests, we identified five SNPs each of which was associated with an individual phenotype; none of these five SNPs was associated with two or more phenotypes. Thus, the marginal tests were limited in terms of finding pleiotropic genes associated with multivariate phenotypes. In contrast, using the proposed multivariate method, we identified two SNPs associated with the four phenotypes together and one of these two SNPs (*rs*11157640) was also found to be associated with ons_reg_drink in the marginal test. Hence, combining multiple phenotypes not only can increase the power of identifying SNPs that may not be identified by marginal tests but also can provide insight into the pleiotropic genes contributing to the common risk expressed by multivariate phenotypes.

## Discussion

Although the simulation shows that adding principal components as covariates to the linear mixed model did not substantially improve the accuracy of estimating the correlation between phenotypes, it can adjust for potential population structure and cryptic relatedness in GWAS as well as improve the estimation of marginal genetic effects [15]. More research is needed to study the optimal number of principal components to be added to the proposed model. Moreover, the simulation study did not consider negative correlations between multivariate phenotypes because the situation is rare in practice. Nevertheless, previous studies have demonstrated that multivariate methods such as MANOVA [33] and MultiPhen [34] tend to have higher power to detect a pleiotropic gene in such a situation. Furthermore, in this study, we only considered *continuous* multivariate phenotypes. Future studies may extend the methodology work to the case of correlated *discrete* phenotypes. For example, in the substance abuse field, many outcomes are zero-inflated count data [35] or ordinal data [36]. A future direction that is particularly challenging is how to analyze multivariate phenotypes with different measurement scales.

## Conclusions

In this study, we propose a new multivariate method for GWAS when multivariate quantitative phenotypes are used to indicate the risk for a complex disease and the data are collected from related subjects. Our approach is a two-step approach. The first step models the association between the genotype and marginal phenotype using a linear mixed model. The linear mixed model uses a random effect to account for the relatedness between subjects. We also extend the linear mixed model by adding principal components as covariates to adjust for potential population structures. Since the sample size in GWAS generally reaches thousands and a certain population structure exists within the subjects, the benefit from adjusting for population structures out-weights the loss of ten degrees of freedom in the linear mixed model. The linear mixed model in the first step also has the flexibility to add demographic variables or other confounding variables to improve precision of estimation. The second step of the proposed method uses the correlation between residuals of the linear mixed model to estimate the null distribution of the Fisher combination test statistic.

The simulation results show that our proposed method controls the type I error rate and is more powerful than the marginal tests across different population structures (admixed or non-admixed) and relatedness (related or independent). The proposed method is also computationally efficient. The first step takes advantage of the efficient program GCTA to carry out marginal tests under linear mixed models. In practice, a few hours are sufficient to derive all marginal *p*-values. The second step only takes a few minutes to compute the Fisher combination test statistic and its null distribution using R software. Furthermore, the real data analysis on the SAGE database demonstrates that applying the multivariate association test may facilitate identification of the pleiotropic genes contributing to the risk for alcohol dependence commonly expressed by the four correlated phenotypes.

## Abbreviations

Not: applicable.
